# Wandering tales: evolutionary origins of mental time travel and language

**DOI:** 10.3389/fpsyg.2013.00485

**Published:** 2013-07-29

**Authors:** Michael C. Corballis

**Affiliations:** Department of Psychology, University of AucklandAuckland, New Zealand

**Keywords:** default mode network, evolution, gesture, hippocampus, language, mental time travel, mind wandering, Pleistocene

## Abstract

A central component of mind wandering is mental time travel, the calling to mind of remembered past events and of imagined future ones. Mental time travel may also be critical to the evolution of language, which enables us to communicate about the non-present, sharing memories, plans, and ideas. Mental time travel is indexed in humans by hippocampal activity, and studies also suggest that the hippocampus in rats is active when the animals replay or pre play activity in a spatial environment, such as a maze. Mental time travel may have ancient origins, contrary to the view that it is unique to humans. Since mental time travel is also thought to underlie language, these findings suggest that language evolved gradually from pre-existing cognitive capacities, contrary to the view of Chomsky and others that language and symbolic thought emerged abruptly, in a single step, within the past 100,000 years.

## Introduction

If people are left to think for themselves undisturbed, without focusing on the immediate environment or on a particular task, their minds wander. Brain-imaging studies show that mind-wandering activates a widespread network in the brain, first identified and described by Raichle et al. (2001) as the *default mode network*, in which the frontal and parietal lobes play a major role. Rather paradoxically, this network is revealed by reverse subtraction; that is, the activation during involvement in some designated task is subtracted from that under passive conditions in which subjects were given no explicit instructions, and were free to let their minds wander (Buckner and Vincent, 2007). Indeed, blood flow to the brain under passive conditions is only about 5–10 percent lower than to the engaged brain, and covers wider regions of the brain. It has been estimated that people spend just under half their waking hours in mind wandering (Killingsworth and Gilbert, 2010).

A critical component of mind wandering is memory, which provides the basic elements from which our mind wanderings are constructed. Memory itself can be divided into declarative memory, which can be made explicit or conscious, and non-declarative memory, which comprises the non-conscious products of learning, such as habits or learned skills like driving or playing the piano. Declarative memory, in turn, can be divided into episodic memory, which is personal memory for past episodes, and semantic memory, which is basic knowledge about the world (Squire, 2004). According to Tulving (1972), episodic memory is unique to humans.

Memory, both episodic and semantic (Klein, 2013), provides the ingredients for imagining possible future events. What has been termed episodic foresight (Suddendorf, 2010), along with autobiographic memory and theory of mind, also makes up much of our mind wandering (Spreng and Grady, 2009), as we preview some future activity or consider possible future options in order to select appropriate action. The capacity to mentally relive past events and imagine possible future ones comprises has been termed *mental time travel* (Suddendorf and Corballis, 1997, 2007), taking us into an imagined future as well as into an imagined past. Both are essentially constructive processes. Brain imaging shows considerable overlap in brain activation between the two, with slightly more frontal-lobe activity in imagining the future (e.g., Addis et al., 2007). Critical to both is the hippocampus, whose role is discussed in more detail below.

## Is mental time travel unique to humans?

He said “What's time? Now is for dogs and apes! Man has Forever!”

—Robert Browning, *A grammarian's funeral*

Extending Tulving's conjecture, Suddendorf and Corballis (1997, 2007) suggested that mental time travel, like episodic memory, is uniquely human. This suggestion, though, has proven contentious. A serious challenge has come from studies of a number of non-human species, including birds. For instance, scrub jays can recover cached food on the basis not only of where it was cached, but also of when it was cached, which might be taken to imply episodic memory of the caching episode itself (e.g., Clayton et al., 2003). Jays also appear to select food to cache based not on present hunger, but on the basis of what they expect to have access to on the following day (Correia et al., 2007). Chimpanzees have been shown to select tools for future use (Osvath and Osvath, 2008) or to collect and conceal stones to be later thrown at visitors to the zoo (Osvath, 2011). In these and other studies there are methodological issues, and questions as to whether the results can be interpreted in terms of associative learning rather than the imagining of past or future events (see Suddendorf and Corballis, 2007 for a critique).

One problem in documenting mental time travel in non-human species is their lack of language. In humans, we have immediate evidence for both episodic memory and future thinking by simply asking for verbal report. Indeed, language itself may have evolved precisely to allow communication about the non-present (Corballis, 2009; Gärdenfors and Osvath, 2010), so we can share our mental travels to other places and other times. The absence of articulate language in non-human species may therefore be considered evidence of incapacity for mental time travel itself. Recent evidence from neurophysiology, though, suggests that non-human animals may indeed have the capacity for at least limited mental time travel, even though they do not have the means to communicate it. A default mode network homologous to that in humans has been identified in the monkey (Vincent et al., 2007), and does suggest a basis for mind wandering, if not for mental time travel itself. More critical, though, may be the hippocampus, which performs two important roles in mammals, as well as in birds.

First, the hippocampus contains so-called “place cells” that encode where an animal is located in space, and so constitute what O'Keefe and Nadel (1978) called a “cognitive map.” This role appears to apply to humans as well as to other mammalian species (Maguire et al., 1998). For example, London taxi drivers, who are required to memorize the streets of London in sufficient detail to navigate without referring to a map or GPS, have enlarged hippocampi relative to controls—although not all trainees manage to finish the course and these show no structural change (Woollett and Maguire, 2011). The taxi drivers also have larger hippocampi than do London bus drivers, who drive on designated routes that impose relatively small demands on memory (Maguire et al., 2006). Similarly, birds that cache items of food in multiple locations, and later retrieve them, have larger hippocampi than birds that do not cache (Macphail, 2002).

Second, the hippocampus appears to be critically involved in declarative memory systems and, in humans at least, in mental time travel generally. Loss of hippocampal function in humans results in severe amnesia, including an apparent inability to imagine possible future events as well as failure to recall past ones (Hassabis et al., 2007a,b; Andelman et al., 2010; Race et al., 2011). Conversely, the hippocampus is activated in neurologically intact individuals when they bring to mind past episodes and imagine possible ones As suggested earlier, the hippocampus appears to be the hub of the system, drawing detailed information from other regions of the brain, including the default-mode network (Addis et al., 2007), for the reconstruction of past or future events. There is some differentiation along the long axis of the hippocampus, with the posterior hippocampus more involved in storage and the retrieval of past episodes and the anterior hippocampus more activated by the imagining of future ones (Szpunar et al., 2007; Martin et al., 2011).

Micro-electrode recordings suggest that the hippocampus may play a similar role in rats. Place cells in the rat hippocampus, which encode specific locations in a structured environment, such as a maze, also fire when the animal is outside that environment, sometimes when the animal is asleep (Wilson and McNaughton, 1994) and sometimes when it is awake but immobile (Karlsson and Frank, 2009). Recordings show that this firing occurs in what have been termed *sharp-wave ripples*, sweeping out trajectories corresponding to earlier locations in the environment. These ripples are accompanied by widespread activation in the cerebral cortex, along with inhibition of activity in the diencephalon, limbic system, and brain stem, suggesting an interaction between hippocampus and cortex in the consolidation of acquired awake experience (Logothetis et al., 2012). It might also be interpreted as representing the experiencing of trajectories, either previously experienced or planned (Corballis, 2013)—in other words, mental time travel.

This is further suggested by evidence that the trajectories need not correspond to actual trajectories that the animal took while it was in the environment. Sometimes they correspond to a previously taken path in a maze, but sometimes to the reverse of such paths, or even to paths through regions the rat did not actually visit (Gupta et al., 2010). This might be taken as evidence for mental time travel along not only past trajectories, but also along imagined future ones. More direct evidence that hippocampal activity signals future behavior comes from rats trained to alternate left and right turns at a particular location in a maze. Between trials, they were introduced to a running wheel, and while they were running differential activity in the hippocampus signaled which turn they would take next. Based on this and other findings, the authors concluded that self-organized activity in the hippocampus, “having evolved for the computation of distances, can also support the episodic recall of events and the planning of action sequences and goals” (Pastalkova et al., 2008, p. 1327).

A similar conclusion is suggested by a more recent study in which rats were given experience with 36 locations in an open-field environment, and learned that a particular goal location contained a reward. When located in randomly chosen locations, the rats were able to determine routes leading back to the goal, even though these routes had not been previously traversed. Sharp-wave ripples pre-played these routes prior to the animal actually setting out (Pfeiffer and Foster, 2013). The authors suggest that the hippocampus “function in multiple conceptual contexts: as a cognitive map in which routes to goals might be explored flexibly before behavior, as an episodic memory system engaging in what has been termed ‘mental time travel’ … ” (p. 5).

The trajectories implied by the hippocampal sharp-wave ripples are much more rapid than those actually taken by the animal. Diba and Buzsáki (2007) recorded hippocampal firing while rats ran back and forth along a straight track. Before each run, the ripples indicated a forward “preplay” of the next run, and after each run a second bout of ripples indicated a “replay” of the run in reverse order. These events were an order of magnitude faster than the sequence recorded during the run itself. While this may suggest that the ripples are not evidence of mental time travel, I suspect our own mental time travels are also speeded up. It takes me an hour to walk from my home to where I work, but mentally it takes less than a minute. Diba and Buzsáki suggest that “preplay events may have a role in ‘planning’ upcoming trajectories” (p. 1242).

These findings, together with the role of the hippocampus is human mental time travel, suggest a strong thread of continuity between rat and human. Indeed, mental time travel in the sense of imagined journeys through space may well have evolved very early as a consequence of movement, and the need to be aware of location and to remember and plan movement through space. It is understandable, too, that the hippocampus should play a role in time as well as in space, since movement in time necessarily involved trajectories in space. That is, the hippocampus operates in 4D space-time. Mental travel, moreover, has one property denied actual travel, in that it can reverse time. We can mentally relive the past, and also imagine events in the reverse order of their actual occurrence, and it seems that rats can do so too.

Nevertheless, there can be little doubt that mental time travel in humans is more complex than that in the rat. Darwin (1871) famously wrote that “The difference in mind between man and the higher animals, great as it is, certainly is one of degree and not of kind” (p. 126). Our own recollections of past events and imaginings of future ones are populated by more than just locations. We remember individual people, actions, objects, emotions, and so forth, and these are present in different combinations in different episodes. Of course we do not yet know whether ripples in the rat hippocampus can signal more than location, and in future research it might be useful to add features or other distinctive characters to the environments in which animals are located, and seek markers in later hippocampal recordings. But for the time being, it seems reasonable to suppose that our imaginings of past and future carry a complexity far greater than that experienced by other species.

## Language

As suggested earlier, language may be considered to have evolved so that we can share our mental time travels, and indeed any experience or knowledge not tied to the immediate environment. If mental time travel is indeed unique to our species, this might well explain why language itself is also confined to *Homo sapiens*. But if the origins of mental time travel reach far back in mammalian evolution, and perhaps even to our joint ancestry with birds, then language itself may be considered to have precursors that long preceded the emergence of our species. This notion, though, is sharply contradicted by a contemporary view that language and the thought processes underlying it emerged *de novo* well within the time span of *Homo sapiens*.

Chomsky (2010), for instance, argues that language evolved in a single step, perhaps as a mutation in a single individual, within the past 100,000 years, long after the emergence of *Homo sapiens* some 200,000 years ago in Africa. He writes

Within some small group from which we are all descended, a rewiring of the brain took place in some individual, call him *Prometheus*, yielding the operation of unbounded Merge, applying to concepts with intricate (and little understood) properties (Chomsky, 2010, p. 59).

Chomsky is also clear that the outcome of this rewiring was a new mode of thought, called internal language (I-language), which was not primarily concerned with communication itself. The mapping of I-language onto external language (E-language) was in effect a secondary outcome. The 7000 or so languages of the present-day world are then considered to have their basis in a shared I-language.

The paleoanthropologist Ian Tattersall reaches a similar conclusion as to the abruptness with which language and symbolic thought emerged:

Our ancestors made an almost unimaginable transition from a non-symbolic, nonlinguistic way of processing information and communicating information about the world to the symbolic and linguistic condition we enjoy today. It is a qualitative leap in cognitive state unparalleled in history. Indeed, as I've said, the only reason we have for believing that such a leap could ever have been made, is that it *was* made. And it seems to have been made well *after* the acquisition by our species of its distinctive modern form (Tattersall, 2012, p. 199).

Such views are profoundly at odds with Darwin's theory of evolution by natural selection, and smack of the miraculous. According to Pinker and Bloom (1990), evolution proceeds in small increments rather than in a single “unimaginable” leap. Indeed Darwin himself wrote:

If it could be demonstrated that any complex organ existed, which could not possibly have been formed by numerous, successive, slight modifications, my theory would absolutely break down. But I can find no such case (Darwin, 1859, p. 158).

Chomsky (e.g., 2011, p. 6) has frequently referred to language as “an organ of the body,” so language might indeed be the case that Darwin feared.

The idea that mental time travel has more ancient roots raises the possibility of a more gradual scenario, and one more consistent with Darwinian theory. Mental time travel itself may well have undergone progressive refinement and extension before reaching a level that might support language. Gärdenfors and Osvath (2010) suggest that the critical period was the Oldowan, dating from some 2.6 to about 1.6 million years ago (Plummer, 2004), and defined by the emergence of stone tools. They describe the Oldowan as a “long ranging culture,” characterized by an extension in time and space. The Oldowan hominins ranged over large distances to gain raw materials or to scavenge or slaughter for food, and long time intervals intervened between the manufacture and use of tools. Gärdenfors and Osvath suggest that this heightened the reliance on prospective cognition, which they consider the basis for the subsequent emergence of symbolic communication. The emergence of tools may have added complexity to the activities of these early hominins, and indeed to their mental time travels, creating further selective pressure toward more effective communication.

Perhaps more critical than mental time travel *per se*, though, were the adaptive advantages to be gained by sharing memories and plans with others. Mental time travel, including memory and prospective cognition, has probably long served to the benefit of the individual, but the ability to share has vast potential to enhance experience and increase survival, at both individual and societal levels. The Pleistocene, the epoch that began with the Oldowan, is widely recognized as the period in which hominins came to occupy what has been termed the “cognitive niche” (Tooby and DeVore, 1987), depending on social bonding and enhanced communication for survival in the more exposed and dangerous environment of the African savanna. Social sharing seems to be ingrained in humans in a manner not evident in our closest non-human relatives. Tomasello (2008) notes, for example, that infants point to interesting objects in their environments, not to request them, but to share the experience with those around them. This may be a precursor to language, in phylogeny as well as ontogeny. Chimpanzees, in contrast, rarely point, and when they do the aim is usually to request something out of their reach.

Underpinning social cognition is theory of mind, the capacity to understand what others think or believe. Some 35 years ago Premack and Woodruff (1978) raised the question, “Does the chimpanzee have a theory of mind?” They were themselves equivocal as to the answer, and their question has led to a long and at times bitter controversy. In a review, Call and Tomasello (2008) conclude that the years of subsequent research have shown chimpanzees to have some understanding of the goals, intentions, perceptions, and knowledge of others, but no understanding of the beliefs and desires of others. True theory of mind, then appears to be limited to humans, at least among extant species, and may well have emerged as a critical aspect of what has also been called the “social mind” as it evolved during the Pleistocene (e.g., Forgas et al., 2007).

The incorporation of theory of mind adds a further dimension to mind wandering; as Buckner et al. (2008) put it, “the default network is active when individuals are engaged in internally focused tasks including autobiographical memory retrieval, envisioning the future, and conceiving the perspectives of others” (p. 1). That is, we can wander mentally not only into past and future, but also into the minds of others. This is well illustrated by the human predilection for story-telling, whether through gossip, fiction, or TV soaps.

Indeed, theory of mind can be regarded as a prerequisite for language itself. Grice (1975) pointed out that language depends on inference rather than explicit decoding. In this respect it contrasts with animal communication, which is generally unambiguous, whereas human language, despite its apparent richness, is characteristically ambiguous and imprecise. In order to converse, individuals must understand what is going on in each other's minds, so that each can infer what the other means. As an example of the ambiguity of language, Sperber and Origgi (2010) give the sentence “It was too slow.” This could mean anything from a chemical reaction being too slow, to the decrease in unemployment in France being too slow, to a car being too slow for an anticipated journey—or a sluggish movement in a symphonic production. In uttering such a sentence, the speaker knows what is in the listener's mind, and has no need to elaborate further. She also knows that the listener knows what's in *her* mind. In this sense, conversational language, at least, serves as a series of prompts to guide shared thought.

Disambiguation also involves projection into the future. Through theory of mind, listeners can create an emulation of what a speaker has just said, and use this to predict upcoming words, meanings, and even grammatical categories. This can not only disambiguate upcoming utterances, but also facilitate rapid comprehension and help the listener deal with noisy input (Pickering and Garrod, 2007). In these respects, then, language draws on both theory of mind and mental time travel.

Contrary to the Chomskyan view of language, another ingredient of language that may go well back in primate and even mammalian evolution is symbolic understanding. Great apes and even dogs are easily taught to understand symbols in terms of what they represent. The bonobo Kanzi communicates by pointing at nonrepresentational symbols on a keyboard, and can even obey simple requests conveyed through spoken English (Savage-Rumbaugh et al., 1998). The gorilla Koko is said to use over 1000 signs and to understand and express signed requests, and he too can respond meaningfully to simple requests spoken in English (Patterson and Gordon, 2001). A border collie called Rico has been shown to rapidly acquire the meanings of some 200 spoken English words (Kaminsky et al., 2004). Rico has since been trumped by another border collie called Chaser, who understands over 1000 proper names as verbal referents (Pilley and Reid, 2011). These accomplishments might also be taken to reflect mental time travel, since they often involve reference to non-present objects or actions. For instance, Kanzi might point to a symbol to request a banana, or ask to be tickled, or invite play, and Rico and Chaser demonstrate their linguistic skills by going on request into another room to fetch a designated object.

Some birds, too, may have the capacity to understand symbols. For instance Alex, a gray parrot, evidently understood number symbols as abstract representation of assemblages of real-world objects in much the same way as apes and small children do. According to Pepperberg (2013), moreover, he learned them more in a human-like than an ape-like fashion. Unlike apes and dogs Alex, like other parrots, was able to produce reasonable simulations of human speech.

### Gestural origins, and the switch to speech

The main impediment to language-like communication in apes and dogs is a deficiency not so much in symbolic understanding as in the means to produce symbols intentionally. Kanzi needs an artificially contrived keyboard to communicate his requests, and Rico and Chaser can understand words but have no means of producing them, or of acquiring surrogates such as signed gestures. Non-human species of course do communicate through calls and cries, but these are for the most part outside of intentional control. Even chimpanzees, according to Premack (2007), “lack voluntary control of their voice” (p. 13866). The more likely option for intentional communication in our primate precursors lay in the hands. Premack goes on to write that chimpanzees “could not have speech. But sign language is a possibility, for they do have voluntary control of their hands” (p. 13866). As the examples of Kanzi and Koko illustrate, apes can learn to communicate through gestures, whether based on sign language or on pointing to visual symbols, but their production of symbols is far less proficient than their ability to understand them—as indeed it is also in human infants.

The idea that language evolved from manual gestures has a long history, going back at least to Condillac (1746/1971) in the 18th century, and restated in modern form by Hewes (1973). The gestural theory was boosted with the discovery in monkeys of mirror neurons, so called because they respond both when the monkey makes a grasping movement and when it observed the same movement performed by another individual (Rizzolatti et al., 1988). Mirror neurons are now considered part of a more extensive mirror system, involving regions in the ventral prefrontal cortex, parietal cortex, and superior temporal sulcus (Rizzolatti and Sinigaglia, 2010), and in fact overlapping extensively with the default network. The idea that mirror neurons may underlie the evolution of language has been elaborated by a number of authors (e.g., Corballis, 2002; Arbib, 2005; Rizzolatti and Sinigaglia, 2008). The gestural theory was also boosted by the realization that the signed languages of the deaf are true languages, with full syntactic and semantic properties, albeit based entirely on visible movements of the hands and face (Armstrong et al., 1995; Armstrong, 1999).

The question then is why authors like Chomsky and Tattersall are so insistent that language emerged as a single package within the past 100,000 years. Their reasoning appears to be based at least in part on archeological evidence for what had been termed a “cultural revolution” within the past 100,000 years, characterized by the seemingly abrupt appearance of bodily ornamentation derived from shells, beads, or animal teeth, of sophisticated cave art, and improved technology in tool making. Summarizing this evidence, Mellars (2005) writes:

To describe the Upper Paleolithic revolution in Europe as reflecting preeminently an explosion in explicitly symbolic behavior and expression is in no sense an exaggeration, as most prehistorians would now agree. We are probably on safe ground in assuming that symbolic behavior and expression of this level of complexity would be inconceivable in the absence of highly structured language systems and brains closely similar, if not identical to, our own (p. 12).

Nevertheless, not all prehistorians are in agreement. McBrearty and Brooks (2000) write of the “revolution that wasn't,” suggesting a more gradual rise in technological sophistication from the Middle Stone Age around 250,000–300,000 years ago, and Shea (2011) similarly argues that human technology over the past 200,000 years is characterized by a variability that persists today, rather than by the abrupt appearance of “modern behavior.” Given that our species is estimated to have emerged some 200,000 years ago, it seems unlikely that there was a dramatic rewiring of the brain within the past 100,000 years.

One possibility is that any change in behavioral patterns in our species was the outcome, not of a rewiring of the brain, nor of the “unimaginable transition” declared by Tattersall (2012), but was the outcome of a change in the manner of communication. I suggested above that language may have originated in manual gestures, and of course it persists in this form in the signed languages of the deaf. If this scenario is correct, then, it must have switched to the vocal form that we call speech at some point. Some have argued against the gestural theory on the grounds that it must have required an unlikely transition from a visuo-manual format to an auditory-vocal one (e.g., Burling, 2005; MacNeilage, 2012). However, in my view the transition is better viewed not as a switch of modalities, but rather as a switch in gestural format. In a recent analysis of the neural mechanisms of speech articulation, Bouchard et al. (2011) conclude that their findings “support gestural theories of speech control over alternative acoustic … or vocal-tract geometry theories” (p. 331).

The switch from manual to voiced language was probably gradual, with facial gestures playing an intermediary role. Signed languages include silent movements of the face as well as of the hands. Facial expressions and head movements can turn an affirmative sentence into a negation, or a question. Mouth gestures are especially important, and have been linked to the equivalent of phonology, especially in European signed languages. Explicit schemes for the phonological composition of mouth movements have been proposed for a number of European Sign languages, including Swedish, English, and Italian (Sutton-Spence and Boyes-Braem, 2001). Mouth gestures can serve to disambiguate hand gestures, and as part of more general facial gestures provide the equivalent of prosody in speech (Emmorey, 2002). Gestures of the face and head also accompany normal speech, including raised eyebrows, winking, down-turning the mouth, tilting or shaking the head.

Speech itself can be regarded as a gestural system, comprising movements of the lips, the larynx, the velum, and the blade, body, and root of the tongue (Studdert-Kennedy, 2005). In the course of evolution, then, intentional communication may have evolved from manual gestures, to overt facial gestures, and finally to the largely hidden gestures that comprise speech—although all three forms of gesture remain present in conversation. Speech gestures, although largely contained within the mouth, retain a visible component, as illustrated by the McGurk effect: A syllable (such as *da*) is dubbed onto a mouth saying another syllable (such as *ba*), and people tend to “hear” what they see rather than what was actually voiced (McGurk and MacDonald, 1976). Other studies show the parts of the brain involved in producing speech are activated when people simply watch silent videos of people speaking (Calvert and Campbell, 2003; Watkins et al., 2003). Rhesus monkeys also show dynamic interactions between perceptions of face movements and voicing, mediated by connections between the superior temporal sulcus and auditory cortex (Ghazanfar et al., 2008).

The transition was probably not a dramatic one, since movements of the hand and mouth are coordinated in activities such as eating, and hand movements and mouth movements mutually interact (Bernardis and Gentilucci, 2006; Gentilucci and Corballis, 2006). The introduction of voicing to the gestural repertoire probably also involved modification of neural mechanisms, including a direct connection between the motor cortex and the nucleus ambiguus (a midbrain vocalization center) that seems to be unique to humans (Jürgens, 2002), and that may explain why vocalization is under precise voluntary control in humans but not in chimpanzees. The continuing link between speech and gesture is further illustrated by the fact that people habitually gesture with their hands as they speak. Moreover gestures are in strict synchrony with speaking, implying a common underlying source (McNeill, 1985).

The switch from manual gesture to speech may well have been the change that led to the dominance of *Homo sapiens*, perhaps even leading to the demise of the other large-brained hominins, including the Neanderthals and the recently identified Denisovans. Although we habitually gesture manually while speaking, manual movements can be disengaged, and play no role in communicating by phone or on radio. Speech, then, may be regarded as an early example of *miniaturization*, tucking language output neatly into the mouth. This resulted in increased energy efficiency. Manual language is effortful, requiring considerable expenditure of energy, while the physiological costs of speech are so low as to be nearly unmeasurable (Russell et al., 1998). Speech adds little to the cost of breathing, which we must do anyway to sustain life. Speech also allows communication at night, or when speaker and audience are not in visual contact. More importantly, perhaps, the transition to speech freed the rest of the body for other activities, including the use and manufacture of tools. This compartmentalization and increase in communicative efficiency may well explain the survival and dominance of our species, whether it occurred as part of the cultural revolution or as a more gradual change over the past 200,000 years.

The emergence of speech as the dominant mode may well have enhanced story-telling, and the sharing of cultural myths and legends that do much to bind societies together. Boyd (2009) points out that religious ideas derive their power less from doctrine than from stories, and stories told orally were passed down the generations with remarkable fidelity before the invention of writing. But the invention of writing has also had a profound effect on human culture, as have more recent inventions such as the Internet and cellphone. The story of human progress may well be in large part the story of advances in communication, and the switch from gestural to vocal communication was an early example. The switch, moreover, may have been more a blend from one to the other, and is still arguably incomplete—especially in Italy.

## Conclusions

It is widely held that humans evolved a distinctive mode of thinking, which included language, in a single step within the past 100,000 years. One important aspect of language is that it permits intentional communication about the non-present, allowing people to share their mental time travels—their experiences, plans, and ideas. It has also been argued that the capacity for mental time travel itself is uniquely human, perhaps evolving in concert with language.

Neurophysiological recordings from rat hippocampus raise the possibility that mental time travel may have ancient origins. This casts a different perspective on the nature of cognitive evolution, and suggests an incremental approach more consistent with Darwinian theory. The cognitive underpinnings of language include not only mental time travel, but also theory of mind and the capacity to attach symbols to real-world entities. The evolution of productive language also required an intentional system with sufficient flexibility to produce the requisite variety of outputs to serve as meaningful symbols. In our primate predecessors, such a system was more likely to have been found in the hands rather than the voice, supporting the idea that language evolved from manual gestures.

The critical period for the evolution of language, then, was likely to have been the Pleistocene, when the transition from a forested environment to the more open savanna placed a survival premium on social bonding and the sharing of experiences. The capacity for mental time travel may have been extended to enable longer-term plans and deeper access to the past. As evidence for cognitive enhancement, brain size approximately tripled during the Pleistocene (Wood and Collard, 1999). These developments would have underpinned more effective communication, with the emergence of obligate bipedalism adding to the communicative power of gesture, initially by freeing the hands but also exposing the rest of the body, including the face, as communicative systems.

The emergence of *Homo sapiens* from around 200,000 years ago seems to have marked further cognitive advances. Our species radiated out of Africa to eventually populate most of the globe, while other equally large-brained hominins were driven to extinction. But rather than suppose that this was the result of some unexplained event, perhaps a mutation, a more parsimonious possibility, and one more consistent with Darwinian theory, is that the emergence of *Homo sapiens* saw a gradual shift from a predominantly manual language to a predominantly vocal one. It may have been this shift that freed the hands for the remarkable and ever increasing technological advances since the dawn of our species (Corballis, 2004). Changes in the mode of communication can have profound effects, the most recent example being the invention of the Internet, and as communication and ensuing technology grow more complex they progress in ratchet like fashion, with each advance building on the previous ones.

Language is characterized by what Chomsky (e.g., 2011) has termed “discrete infinity,” the construction of potentially unlimited meanings from finite elements. In his view, this capacity arose in the singular event that created I-language and universal grammar. The alternative is that the generative aspect of language is provided by mind wandering, a capacity that may have ancient origins. The rat that envisages a past or future trajectory may not have infinite options, but does seem able to imagine trajectories not actually experienced. Mental life no doubt gathered more furniture in the course of evolution, as life itself imposed more challenges. The pace changed with the emergence of the bipedal hominins who were our forebears, as they adapted to new habitats, began to develop tools, and formed more complex social structures. Our mental travels are populated for the most part by familiar elements, such as people, things, places, and actions, which combine in different ways to make up our memories, plans, and fantasies. The combinations are more or less unlimited. With this elaboration, it would have been adaptive to share, so that mental as well as physical resources could be distributed, and our hominin forebears could act and plan in groups. Language itself, then, probably did emerge fairly late in hominin evolution. The best guess, I think, is that language, along with other aspects of social life, emerged in the Pleistocene, and not as a sudden cataclysmic event within the time period of our own species.

### Conflict of interest statement

The author declares that the research was conducted in the absence of any commercial or financial relationships that could be construed as a potential conflict of interest.
